# Targeting neuroplasticity: a viewpoint on a future research direction with Parkinson’s disease-related fatigue

**DOI:** 10.3389/fnagi.2025.1503296

**Published:** 2025-03-25

**Authors:** Shijie Hao, Jianpeng Zou

**Affiliations:** ^1^College of Rehabilitation Medicine, Shandong University of Traditional Chinese Medicine, Jinan, China; ^2^Department of Rehabilitation and Physiotherapy, Affiliated Hospital of Shandong University of Traditional Chinese Medicine, Jinan, China

**Keywords:** Parkinson’s disease-related fatigue, non-invasive brain stimulation, exercise therapy, neuroplasticity, brain-derived neurotrophic factor

## Abstract

Parkinson’s disease-related fatigue has an insidious onset and complex pathomechanisms, causing many adverse effects on patients. In clinical practice, Parkinson’s disease-related fatigue has not received sufficient attention, and its early diagnosis and targeted interventions are inadequate. Currently, pharmacological treatments for Parkinson’s disease-related fatigue have limited efficacy and nonpharmacological therapies such as non-invasive brain stimulation techniques and exercise therapy have been shown to have a role in improving Parkinson’s disease-related fatigue. Further studies have revealed that modulation of functional cortical excitability, induction of neuroplasticity changes, inhibition of oxidative stress, improvement of cardiorespiratory fitness, and enhancement of muscle strength may be potential mechanisms of action of non-pharmacological therapies. As relevant research continues to progress, targeted therapy based on the theory of neuroplasticity may become an important therapeutic idea for Parkinson’s disease-related fatigue. This article provides an overview of the diagnosis, etiology, and treatment of Parkinson’s disease-related fatigue, and on this basis proposes a new diagnostic and therapeutic idea of targeting neuroplasticity to improve Parkinson’s disease-related fatigue for clinical reference. Further studies on the pathological mechanisms of Parkinson’s disease-related fatigue are needed in the future to optimize the treatment regimen of Parkinson’s disease-related fatigue to improve the efficacy of treatment for the benefit of patients.

## 1 Introduction

As a common neurodegenerative disease, studies have shown that the prevalence of Parkinson’s disease is about 1–2% in people over 60 years of age (Bloem et al., 2021; Cabreira and Massano, 2019). Patients with Parkinson’s disease can exhibit a wide range of motor and non-motor symptoms, resulting in disruption of daily life and increased socio-medical burden. The focus of Parkinson’s disease research has been on motor symptoms, while non-motor symptoms are under-recognized and under-intervened clinically due to the insidious nature of the symptoms (Kumar et al., 2022). Fatigue is one of the common non-motor symptoms in Parkinson’s patients (Friedman and Friedman, 1993). Relevant studies have found that about half of Parkinson’s patients present with symptoms of fatigue, as evidenced by the presence of significantly reduced energy levels almost every day of the month or for most of the day, or by exhibiting fatigue that is disproportionate to the level of activity (Barone et al., 2009; Siciliano et al., 2018). In Parkinson’s patients fatigue often occurs insidiously and develops before the onset of motor symptoms and may be a major cause of disability (Friedman et al., 2007). The fatigue experienced by patients with Parkinson’s Disease, excluding other causes, is called Parkinson’s Disease-Related Fatigue (PDRF). PDRF is a feeling of exhaustion and weakness without a clear cause that is disproportionate to physical activity, which is chronic and unpredictable (Bruno and Sethares, 2015; Friedman et al., 2016). PDRF can have adverse effects on patients’ exercise capacity, cardiorespiratory fitness, quality of life, and social participation. PDRF can directly affect the aerobic capacity and exercise endurance of Parkinson’s patients, leading to a decline in motor function. In addition, one study showed that PDRF may further exacerbate neuropsychiatric symptoms such as apathy, depression, and anxiety, thus affecting patients’ quality of life (Béreau et al., 2022). Further studies have found that PDRF shows a gradual worsening trend with the progression of the disease (Ongre et al., 2021; Herlofson and Larsen, 2002). A longitudinal cohort study showed that patients with PDRF had a longer duration of illness, higher doses of levodopa medication, and more significant progression of motor symptoms as well as sleep, mood, cognitive, and autonomic dysfunction (Zhou et al., 2023). Moreover, PDRF increases in severity as the disease progresses and can interact with sleep disorders, pain, and psychosomatic problems, thus further decreasing patients’ quality of life (Diaconu et al., 2024). In some ways, the PDRF may be even more destructive due to the above adverse effects. Therefore, early identification and active intervention of PDRF are significant in improving the quality of life and the prognosis of patients with Parkinson’s disease (Heimrich et al., 2023; Mantri et al., 2021).

## 2 Recognition and diagnosis of PDRF

PDRF is not easy to draw attention to because of its insidious symptoms and the fact that it is mainly based on the patient’s subjective feelings. The diagnosis of PDRF is based on the simultaneous fulfillment of the following conditions: a confirmed diagnosis of Parkinson’s disease, the presence of fatigue and related symptoms, and the exclusion of other causes of fatigue (Kluger et al., 2016). PDRF is primarily assessed using questionnaires. The Fatigue Severity Scale (FSS) and The Multidimensional Fatigue Inventory (MFI) are commonly used clinical scales to assess fatigue in patients with Parkinson’s disease. The FSS focuses on assessing the extent and frequency of fatigue and its impact on daily life. The scale consists of nine items, each rated 0–7, and the total score is finally tallied. The total score reflects the fatigue status, the higher the score the greater the fatigue and the greater the impact on life. The MFI consists of 20 items, each rated on a scale of 1–7, and assesses fatigue on various dimensions, including physical, mental, and activity status. One study found that an FSS score of ≥37 and an MFI score of ≥60 had significant diagnostic value for Parkinson’s disease-related fatigue (Huether et al., 2023). PDRF should be distinguished from lethargy, emotional apathy, and depression in Parkinson’s patients. Characterized by energy deficiency and increased effort required for daily activities, PDRF has a different pathomechanism and treatment than other co-morbid symptoms in Parkinson’s patients. Indeed, PDRF is a multidimensional symptom encompassing physical sensations, emotional components, and cognitive involvement (Mantri et al., 2020). Currently, the diagnosis of PDRF relies on scale scores, and further research is needed to confirm the diagnosis of PDRF through relevant objective neurologic tests or markers (Sarmento et al., 2024).

## 3 Etiology of PDRF

The basic pathomechanism of Parkinson’s disease is the death of dopaminergic neurons in the substantia nigra and decreased secretion of neurotransmitters such as dopamine. A double-blind, placebo-controlled crossover study found that levodopa improved physical fatigue in patients with Parkinson’s disease, suggesting that PDRF is associated with reduced dopamine secretion (Lou et al., 2003). Some scholars agree that other non-motor symptoms of Parkinson’s disease such as sleep disorders, depression and anxiety, and affective and cognitive disorders may contribute to PDRF (Nassif and Pereira, 2018; Lin et al., 2024). According to the analysis of relevant research results, Pathological changes in brain networks, neuroinflammation, and autonomic dysfunction are possible causes of PDRF in patients. Pathological changes in brain networks are important clinical features. A clinical study found abnormal functional connectivity of the sensorimotor network system and bilateral precuneus associated with PDRF. Further, it looked to the left posterior central gyrus as a potential target of action for Parkinson’s Disease-Related Fatigue (Shan et al., 2023). Moreover, some scholars believe that the functional defects of the striatal-cerebellar-cerebral cortical network are involved in the pathological process of PDRF (Hou et al., 2022). In addition, abnormal alterations in functional brain regions related to psycho-cognition may also be a potential mechanism for PDRF. A clinical study found a significant correlation between reduced frontal perfusion and fatigue in Parkinson’s disease patients by SPECT (Abe et al., 2000). Further studies revealed that the altered connectivity of the default mode network in the frontal lobe and posterior cingulate cortex may be an important pathologic mechanism for patients with PDRF (Tessitore et al., 2016). Another study suggested that the frontoparietal attention network may mediate PDRF (Liu et al., 2022). Parkinson’s disease is closely related to the dysregulation of neuroimmune and inflammatory responses (Cohen et al., 2024). One study found that elevated levels of inflammatory components in the serum of PD patients may mediate the development of PDRF (Nikitina et al., 2024). A study on the relationship between plasma inflammatory cytokines and fatigue in Parkinson’s disease showed that plasma levels of inflammatory cytokines, such as IL-1β, IL-18, TNF-α, and phosphorylated α-syn, were significantly increased in patients with Parkinson’s disease accompanied by fatigue compared to those without associated symptoms (Wang et al., 2023). Abnormal aggregation of alpha-synuclein in Parkinson’s patients activates toll-like receptor 4, releasing pro-inflammatory cytokines. The binding of pro-inflammatory cytokines to the endothelial cells of the blood–brain barrier can disrupt the blood–brain barrier, and a large amount of pro-inflammatory cytokines enter the brain and inhibit the reuptake of glutamate by astrocytes, which may be a possible mechanism of Parkinson’s disease-associated fatigue (Wang et al., 2021). In addition to the above causes, autonomic dysfunction is associated with PDRF (Bansal et al., 2022; Zhao et al., 2021). Elevated serum homocysteine levels in Parkinson’s patients treated with levodopa can activate the sympathetic nervous system and lead to autonomic-related blood flow disorders (Mendes et al., 2014). In addition, abnormal vasodilation and contraction due to autonomic dysfunction can affect blood pressure and blood distribution, resulting in decreased muscle function and fatigue (Nakamura et al., 2011). Low-intensity resistance training with blood flow restriction can improve autonomic dysfunction and may be an effective form of exercise for PDRF (Bane et al., 2024).

## 4 Treatment of PDRF

**Figure 1:**
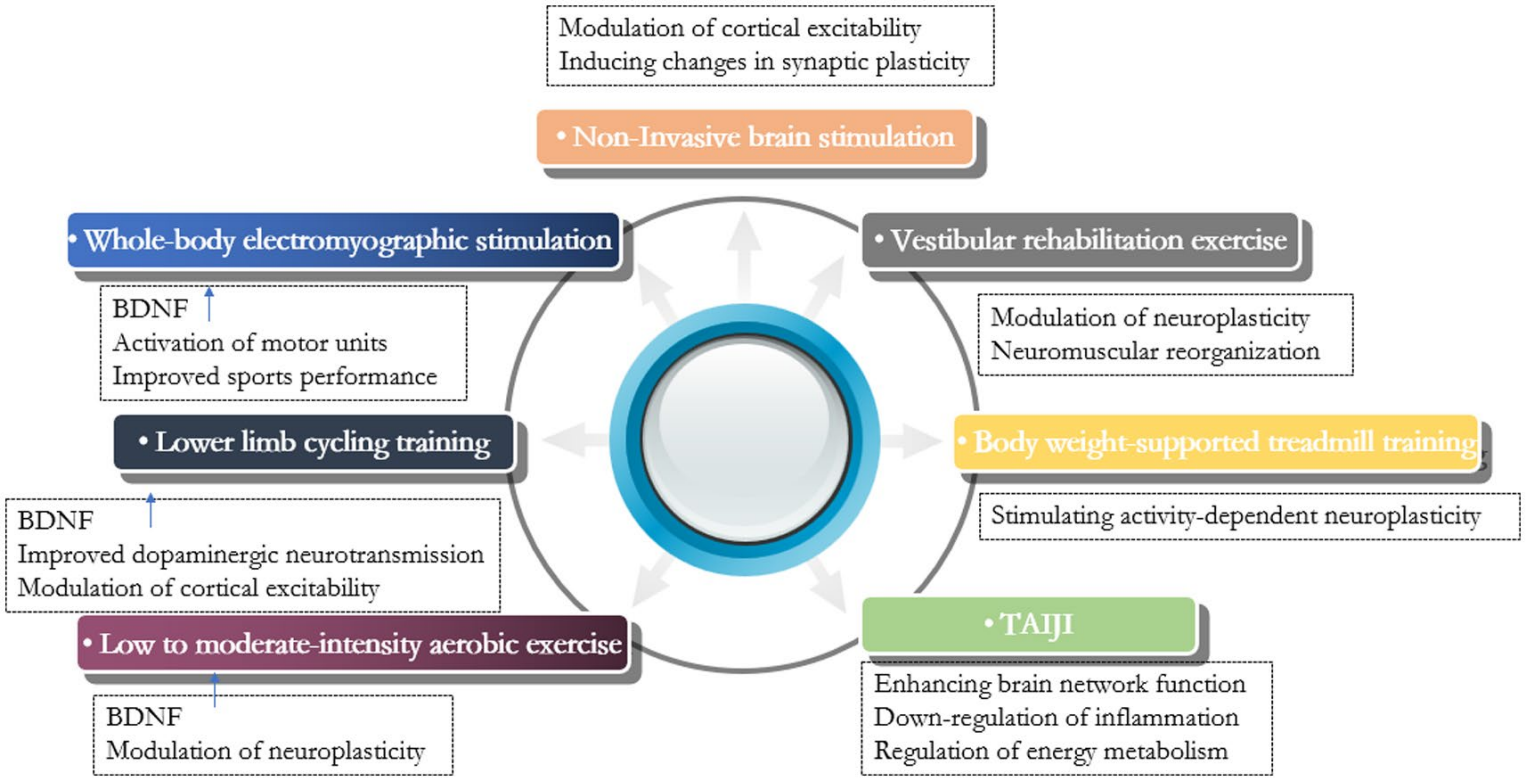
Summary of studies of non-pharmacological therapy for Parkinson’s disease-related fatigue.

In recent years, the understanding and treatment of PDRF have attracted increasing attention, but related progress remains slow. Currently, the treatment of PDRF can be categorized into pharmacological and non-pharmacological treatments, with the former including medications such as levodopa, dopamine agonists, rasagiline, and antidepressants, and the latter including non-invasive brain stimulation techniques, exercise therapy, acupuncture, and yoga. Since the efficacy of pharmacologic treatments is not conclusive, scholars are now turning to nonpharmacologic therapies as important interventions (Figure 1).

### 4.1 Non-invasive brain stimulation

As a non-pharmacological treatment, non-invasive brain stimulation techniques are increasingly used in neurodegenerative diseases (Kourosh-Arami et al., 2021). As a non-invasive brain stimulation technique, transcranial direct current stimulation (tDCS) modulates cortical excitability and induces changes in synaptic plasticity in the cortex of the stimulated area. The current study found that impaired synchronization of interhemispheric resting-state functional connectivity may be one of the neural mechanisms of PDRF (Kourosh-Arami et al., 2021). According to this analysis, the therapeutic concept of modulating altered cortical excitability may help to alleviate patient-related symptoms. Different studies have suggested different sites of action. In addition to the motor cortex, the frontal lobes and the hippocampus may be potential targets for PDRF action. Frontal cortex dysfunction plays an important role in PDRF, and the frontal lobe was chosen as the site of action for tDCS. The efficacy of anodic tDCS stimulation of the frontal lobe in the treatment of fatigue in patients with neurological disorders has been demonstrated. Selecting the dorsolateral prefrontal cortex as the stimulation site may be a potential treatment option for PDRF in the future, and further studies are needed in terms of optimal stimulation parameters and specific efficacy evaluation (Enciu et al., 2011; Azevedo et al., 2022). In addition, the application of anodic tDCS to the hippocampal region, which promotes long-term potentiation and brain-derived neurotrophic factor expression in this region, maybe another option for treating PDRF (Azman and Zakaria, 2022). Simonetta et al. (2023) targeted the primary left motor cortex and gave anodal tDCS stimulation with a stimulation current intensity of 2.0 mA every 20 min and continuous intervention for 10 days to observe the efficacy. The results showed that the NMSS total score and “item 2” (sleep/fatigue) score were significantly reduced in Parkinson’s patients, and the investigators suggested that anodic tDCS stimulation in the M1 region induced changes in cortical neuroplasticity as a potential mechanism for improving PDRF. Other scholars have suggested that high-frequency repetitive transcranial magnetic stimulation also has potential application to promote Parkinson’s disease patients neuroplasticity. Giving high-frequency magnetic stimulation to the corresponding functional brain areas can improve the motor symptoms of patients, as well as the non-motor symptoms such as depression and bad mood, but the selection of the target area and the related parameter settings still need to be further researched (Chen and Chen, 2019; Bougea, 2024).

### 4.2 Exercise therapy

Exercise therapy is an important component of Parkinson’s disease management. Exercise elevates the level of neurotrophic factors, promotes the expression of anti-inflammatory cytokines, activates microglia, and decreases the level of pro-inflammatory cytokines (Svensson et al., 2015). It has been suggested that exercise therapy increases the level of brain-derived neurotrophic factor (BDNF) to regulate neuroplasticity, an important mechanism to improve the clinical outcome of Parkinson’s disease patients (Kaagman et al., 2024). A range of exercise therapies are applied to the treatment of PDRF. di Cagno et al. (2023) observed the effect of whole-body electromyographic stimulation on fatigue in Parkinson’s patients and found that the increase of exercise endurance and coordination was more obvious in patients of the low-frequency whole-body electromyographic stimulation aerobic training group, and its improvement of central fatigue was more significant. They suggested that the elevated serum BDNF levels in patients after aerobic exercise may be an important reason. They also concluded that whole-body electromyographic stimulation can rapidly activate motor units and alter muscle physiological recruitment patterns, effectively improving sarcopenia. Lin et al. (2022) found that Lower Limb Cycling Training significantly improved central fatigue in Parkinson’s patients, and that exercise elevated BDNF levels and improved dopaminergic neurotransmission, as well as altered cortical excitability, maybe the underlying mechanisms. Wu et al. (2021) observed the effects of home-based exercise on motor and non-motor symptoms in Parkinson’s patients and found that low- to moderate-intensity aerobic exercise can significantly improve non-motor symptoms such as fatigue. Home-based exercise is safe and easily accepted by patients. Abasi et al. (2020) found that Vestibular rehabilitation exercise improved fatigue and activities of daily living in Parkinson’s patients, and they identified neuroplastic changes and neuromuscular reorganization as the underlying mechanisms. Atan et al. (2019) concluded that body weight-supported treadmill training stimulates activity-dependent neuroplasticity, reduces exercise resistance, and increases aerobic exercise intensity, which may improve fatigue symptoms in Parkinson’s patients. Another study on the effects of tai chi on non-motor symptoms of Parkinson’s disease and related mechanisms showed enhanced brain network function, down-regulation of inflammation, and enhanced energy metabolism in Parkinson’s patients after tai chi training (Li et al., 2024). Tai chi can be one of the optional forms of exercise for PDRF.

## 5 Targeting neuroplasticity: a new idea for the treatment of Parkinson’s-related fatigue

Neuroplasticity is a fundamental characteristic of the brain. As a self-adjustment mechanism of the human body to cope with changes in the internal and external environments, neuroplasticity is of great significance for neural development and injury repair (Johansson et al., 2020). As a fundamental property of the human brain to adapt to internal pathological damage and external environmental changes, neuroplasticity changes involve different molecular, cellular, and cortical tissue reorganization levels. The essence of neuroplasticity is the self-regulatory mechanism of the brain through structural and functional changes in response to chronic stress, injury, etc. (Zhang et al., 2018). Neuroplasticity is fundamental in neurodegenerative diseases such as Parkinson’s. In the face of the death of related neurons, how the surviving neurons adapt to this change, and compensate the neural network by adding new connections or accelerating neurotransmitter transmission, is significant for delaying the progression of the disease, and improving the related symptoms (Chen and Zhang, 2023). Non-invasive brain stimulation technology and exercise therapy are important therapies for regulating neuroplasticity. Among the non-invasive brain stimulation techniques, tDCS is mostly applied to PDRF treatment, and its specific action targets and parameters, etc. need to be continued to be researched to realize precise rehabilitation. Currently, the theory of exercise-induced neuroplasticity is attracting the attention of scholars. Summarizing the current relevant studies, exercise-induced neuroplasticity may be an important mechanism for the improvement of PDRF in patients.

### 5.1 Exercise protects dopaminergic neurons through activation of astrocytes

The pathophysiologic mechanisms of PDRF are not clear, and dopamine dysfunction may be one of them. Elevating dopamine levels in the central nervous system by dopaminergic drugs may be an important strategy for treating PDRF (Lazcano-Ocampo et al., 2020; Fu et al., 2016). In addition, the use of certain treatments to delay the degeneration of dopaminergic neurons in the substantia nigra is also one of the therapeutic ideas for PDRF. Physiologically, nigrostriatal dopaminergic neurons degenerate slowly with age, and Parkinson’s disease can develop if more than 50% of the neurons degenerate rapidly, with a prevalence of approximately 2–5% according to studies (Morales et al., 2021). Degeneration of nigrostriatal dopaminergic neurons usually begins at striatal synapses, and the resulting proteins and organelles accumulate in degenerating axons and are stored in the globus pallidus, which produces autophagosomes with some autophagy but cannot completely remove the associated cellular debris, which needs to be further engulfed and degraded by astrocytes (Morales et al., 2020). Astrocytes provide the optimal microenvironment for maintaining neuronal function and survival (Valori et al., 2019; Kuter et al., 2020). Studies have shown that dysregulation of glial cell phagocytosis and degradation and altered microenvironment of dopamine neurons is one of the pathological mechanisms of Parkinson’s disease (Tremblay et al., 2019). In the central nervous system, astrocytes can actively participate in the construction of synaptic homeostasis by phagocytosis of synapses, neuronal fragments, axonal mitochondria, and pathological protein aggregates. In addition, astrocytes may also regulate microglia phagocytosis by secreting molecules such as IL-33 and C3 (Lee and Chung, 2021). If astrocytes do not provide the lysosomes required to complete the degradation of dopaminergic neuronal fragments, the degenerated fragments may activate microglia in the medial forebrain tract near the dopaminergic axons, leading to neuroinflammation and the spread of retrograde axonal degeneration to dopaminergic neurons in the substantia nigra (Tagliaferro and Burke, 2016). Thus, the phagocytosis of astrocytes is essential to stop the activation of microglia and the spread of retrograde axonal degeneration in Parkinson’s pathological process (Ho, 2019). Therefore, enhancing the phagocytosis of astrocytes through exercise activation can effectively delay the progression of the disease, which is a possible mechanism for the rehabilitative efficacy of exercise therapy when applied to Parkinson’s disease. Other studies have suggested that the dysfunction of astrocyte homeostasis leading to neuronal excitotoxicity in patients with Parkinson’s disease is the key to its progression, and the main mechanism lies in the impaired ability of glial cells to reuptake glutamate. Increased levels of glutamate, an excitatory neurotransmitter, in the central nervous system lead to abnormal synaptic signaling, causing neuronal excitotoxicity and death (Iovino et al., 2020). After exercise, astrocytes have an increased capacity to take up glutamate and further convert glutamate and γ-aminobutyric acid to glutamine, which both eliminates the continued adverse effects of the relevant neurotransmitters and provides material support for neuronal synthesis of the relevant transmitters (Zhou et al., 2019). In addition, astrocytes secrete antioxidant factors (glutathione and ascorbic acid) during exercise to protect neurons in the central nervous system of patients with Parkinson’s disease (Isooka et al., 2021).

### 5.2 Exercise elevates BDNF levels to regulate neuroplasticity

Nigrostriatal neuronal degeneration and altered striatal plasticity are important pathogenetic mechanisms in Parkinson’s disease. BDNF is a key factor in neuronal development, survival, and is essential for the maintenance of neuronal function in the striatum and substantia nigra. Studies have shown that BDNF promotes the survival of dopaminergic neurons, maintains the functional activity of striatal neurons, and improves dopamine production and uptake through the BDNF/TrkB signaling pathway (Ali et al., 2024; Urbina-Varela et al., 2020; Hernández-Vara et al., 2020). In addition, BDNF is involved in the regulation of neuroplasticity by activating the tropomyosin receptor kinase B (TrkB) and inducing long-term potentiation effects in the striatum (Wolf et al., 2024). BDNF has neuroprotective effects and is involved in the regulation of neuroplasticity, so it is closely related to the onset and development of neurodegenerative diseases (Koyya et al., 2024). Relevant studies have found that BDNF synthesis is affected by a variety of factors, and neurodegenerative diseases, aging, as well as chronic inflammatory and stressful stimuli can lead to a decrease in its synthesis (Molinari et al., 2020). A meta-analysis showed that BDNF levels were significantly lower in PD patients compared to healthy controls (Chen and Zhang, 2023). From neuropathological analysis, BDNF is involved in the regulation of neuropathological processes such as apoptosis, mitochondrial dysfunction, and oxidative stress injury, so it may be a potential target of action for PD management.

In Parkinson’s patients, exercise can be neuroprotective by promoting the release of growth factors (Schaeffer et al., 2022) and it is an effective way to induce changes in neuroplasticity (El-Sayes et al., 2019). Clinical studies have found that aerobic exercise increases volume and functional activity in the hippocampal areas of the prefrontal and temporal cortex, the latter two of which are strongly associated with fatigue in patients with Parkinson’s disease (Abe et al., 2000; Zhang et al., 2018; Chou et al., 2016). In addition, aerobic exercise may slow down the progression of Parkinson’s disease and Improve fatigue and related symptoms in patients through BDNF and its signaling pathway (Johansson et al., 2020). Both prolonged aerobic exercise and a single session of light to moderate intensity exercise increase serum levels of BDNF in patients with PD (Azevedo et al., 2022; El-Sayes et al., 2019). BDNF plays an important role in the process of neurogenesis, neuron survival, proliferation, and differentiation (Azman and Zakaria, 2022). The main mechanism is that mature BDNF can bind to the TrKB receptor, activate signaling pathways such as MAPK, PLC-γ, PI3K/Akt, and then activate transcription factor cAMP response element-binding protein, playing roles in anti-apoptosis and synthesis of cytoskeletal proteins (Palasz et al., 2020). Another study found that BDNF also protects neurons and delays the progression of Parkinson’s disease by promoting Signal transducer and activator of transcription 3 (STAT3) phosphorylation and regulating neuronal autophagy (Geng et al., 2023). In conclusion, aerobic exercise induces structural and functional changes in relevant brain regions through BDNF and its signaling pathways (Kim et al., 2021; Dou et al., 2022), thus serving to improve the symptoms of Parkinson’s patients. In addition, aerobic exercise induces the release of BDNF, enhances synaptic GABA clearance, promotes the development of neuromyelin and damage repair, and thus alleviates central fatigue in Parkinson’s patients. In addition, inhibition of oxidative stress, promotion of growth factor release, and repair of mitochondria to regulate energy metabolism may also be mechanisms by which aerobic exercise improves fatigue.

## 6 Summary and outlook

As one of the common non-motor symptoms of Parkinson’s disease, PDRF has many adverse effects on patients. A related study found that more than half of PD patients in Turkey had problems with sleep, fatigue, mood, cognition, and urinary tract (Onder and Comoglu, 2024). PDRF is a multidimensional clinical condition associated with factors such as physical sensations, emotional changes, and cognition, and patients present with subjective high levels of distress. PDRF significantly impacts patients’ daily lives, and exploring its underlying pathophysiological mechanisms for targeted interventions is a direction for future research (Tinazzi et al., 2025). The specific etiology and pathogenesis are still in the exploratory stage, but it is imperative to understand PDRF based on biological, psychological, and social models (Weis, 2024). In recent years, studies on PDRF have attracted increasing attention from scholars. In the early recognition and diagnosis of PDRF, there are many problems with the current subjective scale assessment, and exploring possible biomarkers has become a hot research topic (Stocchi et al., 2024; Popescu et al., 2024). Mass spectrometry imaging (MSI), a novel molecular imaging technique that visualizes the distribution and content of molecular compounds, may be an early screening tool for non-motor symptoms in PD patients (Lai et al., 2024). In addition, exploring changes in serum biomarker levels with the help of relevant techniques may provide clinical implications for the early diagnosis of PDRF. It has been suggested that serum insulin-like growth factor 1 is correlated with non-motor symptoms in patients with Parkinson’s disease and may be a potential biomarker for PDRF (Gu et al., 2025). Treatment for PDRF is another topic of current research. Pharmacologic therapy is based on levodopa, dopamine agonists, rasagiline, and antidepressants, and the efficacy is not exact. Non-pharmacologic therapies have some potential to improve PDRF and deserve further in-depth study. From the current study, modulating functional cortical excitability and inducing changes in neuroplasticity may be important therapeutic concepts for PDRF (Andica and Kamagata, 2024; Jahan et al., 2024). In the future, in addition to continuing to study the pathomechanisms of PDRF, targeted development of non-pharmacological therapies that target neuroplasticity and functional cortex from the center may be the key to improving PDRF (Popescu et al., 2024; Sharbafshaaer et al., 2024).

## Data Availability

The original contributions presented in the study are included in the article/supplementary material, further inquiries can be directed to the corresponding author.
